# Intricate Networks in Nomenclature: Cases of Naming in *Arthrocaulon*, *Arthrocnemum*, and *Salicornia* (Amaranthaceae)

**DOI:** 10.3390/plants13131783

**Published:** 2024-06-27

**Authors:** Duilio Iamonico, Ib Friis, Mauro Iberite

**Affiliations:** 1 Department of Environmental Biology, University of Rome Sapienza, Piazzale Aldo Moro 5, 00185 Rome, Italy; mauro.iberite@uniroma1.it; 2 Natural History Museum of Denmark, Zoological Museum, Universitetsparken 15, DK-2100 Copenhagen Ø, Denmark; ibf@snm.ku.dk

**Keywords:** *Arthrocaulon*, *Arthrocnemum*, Delile, Forsskål, new synonym, *Salicornia*, *S. fruticosa*, *S. glauca*, *S. virginica*, *Sarcocornia*, typification

## Abstract

The nomenclatural status and typification of the names *Arthrocaulon macrostachyum*, *Salicornia fruticosa*, *S. fruticosa* var. *deflexa*, *S. fruticosa* var. *glaucescens*, *S. fruticosa* var. *intermedia*, *S. fruticosa* var. *humilis*, *S. fruticosa* var. *pachystachya*, *S. fruticulosa*, *S. glauca*, *S. lignosa*, *S. macrostachya* var. *virescens*, *S. macrostachya* var. *glaucescens*, *S. perennis*, *S. radicans, S. radicans* var. *caespitosa*, *S. sarmentosa*, *S. sempervirens*, and *S. virginica*, as well as an unnamed β-variety of *S. fruticosa* proposed by *A. Bertoloni*, are investigated. Concerning *A. macrostachyum*, we document that the type indicated in literature (G00177362) is not a holotype, and that lectotypification is necessary. A specimen from G (G00687638) is here designated as a lectotype. On the level of variety, *Arthrocnemum fruticosum* var. *macrostachyum* is an earlier legitimate name for *Salicornia fruticosa* var. *pachystachya*. Furthermore, Piirainen et al. are incorrect when citing Forsskål’s “Salicornia” from Alexandria as “S. virginica Forssk.”; it is not a new name and should be cited as S. virginica auct. non L., as published in Forsskål’s *Flora Aegyptiaco-Arabica*. Like with numerous other parallel cases in *Flora Aegyptiaco-Arabica*, Forsskål’s designation of “Salicornia virginica” for an Arabian plant is to be considered a misapplication of the earlier Linnaean name for an American plant. *Arthrocnemum glaucum* (a nomen illegitimum of Ungern-Sternberg), was listed as type species of Arthrocnemum by the Names in Current Use project; the basionym, *Salicornia glauca* Delile, is here lectotypified and identified as Arthrocaulon meridionale, published by Ramirez et al. Updated synonymies of *Arthrocaulon macrostachyum*, *A. meridionale*, *Salicornia fruticosa*, and *S. perennis* are proposed. *Salicornia sempervirens* is an invalid name according to Art. 36.1a of ICN. No original material was found for *S. radicans* var. *caespitosa*. This paper also refer to lecto- or neotypifications on specimens deposited at BM, G, LINN-HS, LY, MPU, NAP, and PAL, and their current taxonomic positions are suggested in a taxonomic part of the paper.

## 1. Introduction

The subfamily Salicornioideae Ulbr. (Amaranthaceae Juss.) was investigated in detail by Kadereit et al. [1] and Piirainen et al. [2] through the analysis of molecular sequences. Both studies showed that the traditional circumscription of most genera is supported, except for the clades *Halosarcia*/*Tecticornia* and *Sarcocornia*/*Salicornia*. The accessions around *Arthrocaulon macrostachyum* (Moric.) Piirainen & G.Kadereit (≡*Salicornia macrostachya* Moric.), form a well-supported clade, which is basal to a large group including further four well-supported clades (“Salicornia/Sarcocornia”, “Tecticornia”, “Arthrocnemum subterminalis”, and “Microcnemum”; see Piirainen et al., 2017 [2]). According to these authors, *Arthrocaulon* Piirainen & G.Kadereit, comprises two species, i.e., *Arthrocaulon macrostachyum* (Moric.) Piirainen & G. Kadereit, distributed in the Mediterranean Basin, NE- and NW-Africa, Macaronesia and W-Asia, and *Arthrocaulon franzii* (Sukhor.) Piirainen & G.Kadereit from the from Cape Verde. More recently, Ramírez et al. [3] (p. 34) described a third species (*Arthrocaulon meridionale* [as ‘*meridionalis*’] Est.Ramírez, Rufo, Sánchez Mata, V. Fuente) from Sicily. This is also said to occur in Sardinia (South Italy), Spain (Melilla), Turkey and Iran, where it was previously identified as *A. macrostachyum*. Note that Piirainen and G. Kadereit [2] assigned their new generic name *Arthrocaulon* to be neuter by indicating *A. macrostachyum* (Moric.) Piirainen & G. Kadereit to be its type, with an adjectival epithet in the neuter. Generic names without a taxonomic tradition retain the gender assigned by their author (Art. 62.1 of the Shenzhen Code [4]); thus, new adjectival epithets in *Arthrocaulon* must also be neuter, e.g., *A. meridionale* (ICN Art. 23.5).

The species of *Arthrocaulon* are sometimes still referred to *Arthrocnemum*, for example by de la Castroviejo [5] (p. 526–527), de la Fuente et al. [6] (p. 1249), and Ramírez et al. [7] (on p. 1422 and nearly all following pages), where the typification of the generic name *Arthrocnemum* as *Salicornia fruticosa* (L.) L. by Pfeiffer [8] (p. 279) is rejected. However, Pfeiffer’s typification has the priority of designation (ICN Art. 9.19) and is cited as acceptable according to Art. 7.11, Ex. 17 of the Shenzhen Code [4], thus making *Arthrocnemum* a synonym of *Salicornia* unless that genus is very narrowly defined. For this reason, Piirainen and G. Kadereit published the new generic name *Arthrocaulon*. In this study, we accept *Arthrocaulon* and Pfeiffer’s typification of *Arthrocnemum* (for details of the various typifications of *Arthrocnemum*, see ‘Section 3.1.7 *Salicornia glauca*’ of the present paper).

As a whole, the genera *Arthrocaulon* and *Salicornia* are difficult from the taxonomical point of view due to their low number of morphologic characters, their high phenotypic variability, and recurring hybridization [1,2,3]. This has caused a proliferation of names over time, sometimes leading to nomenclatural problems (see [4] for an example).

As part of the ongoing research on Salicornioideae [9,10,11,12,13], here we present a range of nomenclatural and taxonomic notes regarding names that are used to refer mostly to perennial species belonging to the genera *Arthrocaulon* and *Salicornia*, names which mostly have been previously untypified.

## 2. Material and Methods

This study is based on the analysis of the relevant literature (i.e., protologues of the names investigated and works in which these names have been listed or discussed). It is also based on a search for and the examination of specimens preserved at the herbaria BM, C, CGE, G, E, FI, K, LINN, LINN-HS, LY, MA, MANCH, MNW, MPU, NAP, OXF, P, PAL, RO, SLBI, SWA, and UPS (acronyms following those of Thiers [14]).

Nomenclatural articles and references to the Glossary, as cited throughout the text, are given following the *Shenzhen Code* [4].

The abbreviations of author names follow the guidelines of IPNI (https://www.ipni.org/, accessed on 12 June 2024).

The information shown in the label transcriptions is given between double quotation marks.

The studied names are listed alphabetically according to epithets.

## 3. Results and Discussion

### 3.1. Publication and Typification of the Names

#### 3.1.1. *Salicornia anceps*

Castroviejo [15] (pp. 212–213) discussed Lagasca’s name *Salicornia anceps* [16] (p. 52) and stated that “Typus: Se cría en Roquetas y Cabo de Gata, en donde le encontró don Simón de Rojas Clemente (MA 29474)” (note that Lagasca’s herbarium and types were destroyed, but duplicates are preserved at MA according to the HUH-Index of Botanist [17]). Although the phrase “designated here” (or an equivalent) was not reported by Castroviejo [15], the typification (lectotype available at https://imagenes.rjb.csic.es/herbarioV/visorVCat.php?img=MA-01-00029474, accessed on 20 June 2024) is to be accepted according to Art. 7.11 of ICN (Castroviejo’s statement was published before 1 January 2001). The MA specimen is a sterile and terminal part of one plant and, therefore, it cannot be identified with certainty as any of the *Salicornia* perennial species. This was also the conclusion of Castroviejo [15], who considered this name to be ambiguous. We agree with Castroviejo [15] on this point. Based on our preliminary check, the issue is still quite complicated, and although we accept Castroviejo’s lectotypification, we here decide to postpone the further identification of type. Conclusive identification of the type material might involve designating an epitype, collected from the *locus classicus*.

#### 3.1.2. *Salicornia fruticosa* var. β by Antonio Bertoloni

Bertoloni [18] (p. 18) recognized, under *Salicornia fruticosa*, a variety of β, giving the following diagnosis: “caule humili, subspithameo, inferne decombente, radicante”; since no epithet was proposed, this taxon of Bertoloni’s has no nomenclatural standing. Despite this, clarifying Bertoloni’s detailed studies and concept is useful for understanding the concept of Koch [19] (p. 693) regarding his *Salicornia fruticosa* var. *pachystachya*. In terms of this variety, Koch reported that “… spicis duplo crassioribus: *S. macrostachya* Moricand. fl. venet. 1. 2, sec. Bertol.” Bertoloni [18] (p. 18) listed the following synonyms under his unnamed variety β:

- “*Salicornia fruticosa Bert. Amoen. Ital. p.* 237. *n.* 1”. This is from Bertoloni [20] (p. 237), who accepted the Linnaean *S. fruticosa* as occurring at “... maris Adriaticis littora petii, ubi ex canalis pistrinorum ostio Ravennatem portum versus excurrens hasce plantas ibi nascentes adiveni”. Bertoloni [20] (p. 327) also cited Smith’s *English Botany* [21] and, specifically, the illustration no. 2467 (“*Salicornia fruticosa Sp. p. 5. Engl. bot.* Table 2467”);
- “Salicornia fruticulosa *Tin. Cat. an.* 1827. *p.* 280”. This is from Tineo [22] (p. 280), who described the new species *S. fruticulosa* by providing a diagnosis and the provenance (“*Crescit in inundatis locis maritimis prope Panormum;* a Mondello”; he also stated “*simul cum S. macrostachya*, *et herbacea*” and “Differt a S. fruticosa praesertim caule decumbente, radicante”;
- “S. radicans *Viv. Fl. Lybic. Spec. p.* I* *Ten. Syll. p.* 8. *n.* 4”. This is from, respectively, Viviani [23] (p. 1) and Tenore [24] (p. 8). Both these authors accepted Smith’s *S. radicans* [25]; Viviani [23] specified the number of the table in Smith’s work [25], i.e., Table 1691.

Based on the synonyms and the literature cited by Bertoloni [18] (p. 18), it is clear that the Italian author, with his *Salicornia fruticosa* var. *β*, had in mind to propose a new taxon for prostrate plants occurring in coastal areas of eastern Italy, spanning from the north (“litora Ravennatia, et Venetiis”—Ravenna and Venezia are two cities of, respectively, the Emilia-Romagna and Veneto regions, in NE-Italy) to the south and to the cliffs of Gargano’s promontory [“… rupibus *di Viesti* prope Garganum”, where Vieste is a small town of the Apulia region (SE-Italy) located on the Gargano promontory] and occurring in Sardinia and Corsica (“... *Bonifacio*, et *Portovecchio* .... *Bastia*). To fully understand Bertoloni’s concept of his unnamed variety *β*, we first checked the Herbarium BOLO, where Bertoloni’s herbarium and types are mainly preserved [26]. We found the following four sheets:

- BOLO100045 (two plants), collected “*in pratis prope litora Ravennatia*, *ubi copiosa*” in 1818;
- BOLO100046 (two plants), collected in “*Ex litore Veneto*” (“*misit Rechinger 1824*”);
- BOLO100047 (one plant), collected in “*Scogli di Vieste sul Gargano*” (“*misit Tenore 1828*”);
- BOLO100048 (four pieces of one plant), collected in “*Ex Sardinia*” (“*misit Moris 1828*”).

The identification of the BOLO specimens is not a simple issue since the ranges of the diagnostic characters between the related *Arthrocaulon macrostachyum* and *A. meridionale*, given by Ramírez et al. [7], appear to be partially overlapping and as the reliable diagnostic character is essentially determined by whether the plants are diploid or tetraploid. Therefore, as a valid and workable distinction between these two species, we rely on their chorology. We therefore identify BOLO100045, BOLO100046, and BOLO100046 (both collected from the Adriatic coast of Italy; Emilia-Romagna, Veneto, and Apulia regions, respectively) as *A. macrostachyum*, whereas BOLO100048 (the plant from the Sardinia region) is identified as *A. meridionale*.

Regarding the synonyms cited by Bertoloni [18] (p. 18), we can make the following remarks:

- Smith’s Table 2467 [21] shows a sterile plant. Therefore, it cannot be identified according to the current taxonomic concepts regarding *Salicornia* (see e.g., Ball 2003 [27], Iberite 2018 [28]). However, based on the description given, Smith [21] reports “small, short dense spikes” which is a diagnostic character of *S. perennis* Mill. Therefore, Smith’s concept of Linnaean *S. fruticosa* is actually identical with *S. perennis*;
- Tineo’s *Salicornia fruticulosa* is a name reported as being “unplaced” in POWO [29]. The name appears to be untypified based on our literature search (see discussion under ‘Section 3.1.6 *Salicornia fruticulosa*’).

Thus, with regard to the plants on the Adriatic coast, the synonyms and specimens associated with Bertoloni’s *Salicornia fruticosa* var. β fall within the modern concept of *Arthrocaulon macrostachyum.* When Bertoloni [18] (pp. 17–18) established his taxonomy of *Salicornia fruticosa*, he made it clear that his unnamed var. β was a prostrate plant and that this variety was not identical with *S. macrostachya* by Moricand, a name which he cited as a full synonym of *S. fruticosa*. When Moquin-Tandon [30] (p. 112), earlier than Koch, had established the variety *Arthrocnemum fruticosum* var. *macrostachyum* (Moric.) Moq., he cited “Salicornia macrostachya Moric.!” and “S. arbuscula DC! Herb.” in synonymy (for discussion of the latter, see 3.1.10. *Salicornia macrostachya*), but did not mention Bertoloni’s treatment. When Koch [19] proposed his var. β, which he, unlike Bertoloni, gave an epithet (“var. β. *pacystachya*”) and the synonymy “S. macrostachya Moricand fl. Venet. 1. 2., sec. Bertoloni.”, one might assume that Koch’s “var. β. *pachystachya*” represented a naming of Bertoloni’s “var. β”, but this is not the case; the reference “Moricand 1.2” must refer to Volume 1, part 2 in Moricand’s *Flora Veneta*, where the name *Salicornia macrostachya* Nob. [=Moricand] is proposed. Therefore, when Koch stated that he followed Bertoloni’s work, he must mean that he followed Bertoloni’s treatment of Moricand’s *S. macrostachya* in full synonymy under *S. fruticosa*, and not that he only referred to Bertoloni’s unnamed variety β, although both Bertoloni and Koch have a “var β”. Therefore, Bertoloni’s var. β and Koch’s var. β are different and Koch’s “*S. fruticosa* var. β. *pachystachya*” includes both Moricand’s *Salicornia macrostachya* as a synonym of *S. fruticosa* in the sense of Bertoloni and Bertoloni’s var. β. Because of the existence of the earlier name at the rank of variety [*Arthrocnemum fruticosum* var. *macrostachyum* (Moric.) Moq., proposed by Moquin-Tandon [30] (p. 112), who cites *Salicornia macrostachya* Moric. in synonymy], *S. fruticosa* var. *pachystachya* was a nomenclaturally superfluous and illegitimate renaming and is to be typified by original material of the name *S. macrostachya* (see ‘Section 3.1.5 *Salicornia fruticosa* var. *pachystachya’* and the discussion of synonymy in ‘Section 3.2 Taxonomic treatment’ below).

#### 3.1.3. *Salicornia fruticosa* Varieties by Michele Tenore

Tenore [31] (p. 582) classified *Salicornia fruticosa* into three varieties, namely, var. *glaucescens* Ten. (diagnosis: “elata, caulinibus lignosis, rami patentibus, articuli valde remotis”), var. *intermedia* Ten. (diagnosis: “radicans glauca”), and var. *humilis* Ten. (diagnosis: “virescens, caulibus procumbentibus radicantibus ramisque divaricatis”). No information about these three varieties was found in volume IV of Tenore’s *Flora Napolitana* [31] (p. 5), where *S. fruticosa* was listed with only the var. *macrostachya*. According to Tenore [31] (p. 5), the localities of *S. fruticosa*, and presumably also the localities of the varieties published the following year, were “In inundatis salsis. *Fusaro*, *Maremorto*, *Lago salso*”. We traced two sheets at NAP (where Tenore’s herbarium and type are mainly deposited; see [32]), i.e., NAP0000051 and NAP0000052. NAP0000051 bears three parts of plants and the following two labels: “*Salicornia fruticosa virescens varietas*” and “*Salicornia fruticosa humilis virescens*”. Since the two labels are not clearly associated with any of the three parts of plants, we suppose, according to the diagnosis of var. *humilis*, that the part in the center of the sheet (which includes roots and is clearly procumbent) can be referred to as this variety. The sheet NAP0000052 bears two parts of plants and the label “*Salicornia fruticosa glaucescens* ...|*Fusaro*”, where *Fusaro* is a coastal lake occurring in the Bacoli Municipality (Province of Naples, Campania region, southern Italy), as reported by Tenore [31] in *Flora Napolitana* (see above). Unfortunately, no date of collection was reported in these two NAP sheets and, therefore, we cannot be sure that they are ante-1831 collections. Therefore, we prefer to avoid their use as lectotypes (Art. 9.3 and 9.4 of ICN [4]). Since no further sheet of original material was found, neotypifications are required according to Art. 9.8 of ICN [4]). We designated NAP0000051 (plant part on the center of the sheet) as the neotype of *S. fruticosa* var. *humilis* and NAP0000052 as the neotype of *S. fruticosa* var. *glaucescens*. Finally, as regards var. *intermedia*, we traced two specimens at LY (barcodes LY0517535 and LY0517536). These were useful for neotypification since they were collected at “lago fusaro”, as reported in an original label, and *Pellanda* s.n. (LY0517535) is here designated as a neotype of *S. fruticosa* var. *intermedia*. NAP0000052 is identifiable as *S. fruticosa* (an erect and large branch with many terminal spikes, up to 4 cm long). The other types (NAP0000051, LY0517535, and LY0517536) cannot be identified according to De La Fuente et al. [33]. In fact, based on these authors, NAP and LY specimens can be referred to as *Saronornia perennis* (Mill.) A. J. Scott. (currently *Salicornia perennis*) or *Sarcocornia alpinii* (Lag.) Rivas Mart. (*Salicornia alpinii s.s.* according to [2]), but seeds are lacking in these types. Further investigations (field surveys) are necessary to reach a taxonomic conclusion about these two names that were given by Tenore. As a consequence, we prefer to avoid synonymizing these two names, which are presented separately (see ‘Section 3.2 Taxonomic Treatment’).

#### 3.1.4. *Salicornia fruticosa* var. *deflexa*

Rouy [34] (p. 60) described a form of *Salicornia fruticosa* (L.) L., *S. fruticosa* var*. deflexa* Rouy, which was characterized by “Rameaux tombants ou decombants, radicanta, à extrémité asccendante”; syntypes of this (Art. 9.6 of ICN [4]) were also reported (“Manche: Brévands et Saint-Vaast (*Corbiere*)”).

We traced just one specimen that was part of the original material for *Salicornia fruticosa* var. *deflexa*, i.e., a Corbière’s collection in Saint-Vaast was preserved at LY (barcode LY0745272). This specimen is here designated as its lectotype (Art. 9.12 of ICN [4]). According to the current concept [1,11], LY0745272 is identifiable as *S. perennis*.

#### 3.1.5. *Salicornia fruticosa* var. *pachystachya*

As mentioned in ‘Section 3.1.2 *Salicornia fruticosa* var. *β’* varieties referred to as var. *β* were published by Antonio Bertoloni and Koch [19] (p. 693) under *Salicornia fruticosa* (L.) L., the latter as a name for a variety “β *pachystachya*, spicis duplo crassioribus: *S. macrostachya* Moricand. fl. venet. 1. 2, sec. Bertol.”, and this should not be confused with Bertoloni’s var. β. When Bertoloni [18] (pagg. 17–18) established his taxonomy of *S. fruticosa*, he made it clear that his unnamed var. β was a prostrate plant and that this variety was not identical with *S. macrostachya* Moric., which he cited as a full synonym of *S. fruticosa*. When Moquin-Tandon [30] (p. 112), earlier than Koch, established the variety *Arthrocnemum fruticosum* var. *macrostachyum* (Moric.) Moq., he cited “Salicornia macrostachya Moric.!” and “S. arbuscula DC! Herb.” in synonymy, but did not mention Bertoloni’s treatment (see in ‘Section 3.1.9 *Salicornia macrostachya*’ for details). Koch’s *Salicornia fruticosa* var. β *pachystachya* is therefore an illegitimate renaming of *Arthrocnemum fruticosum* var. *macrostachyum* (Moric.) Moq.

#### 3.1.6. *Salicornia fruticulosa*

Tineo [22] (p. 280) described *Salicornia fruticulosa* Tineo, providing a detailed description and citing its provenance (“*Crescit in inundatis locis maritimis prope Panormum*; a Mondello”).

We traced three relevant specimens at PAL (where Tineo’s herbarium and types are preserved [35]), i.e., PAL58780, PAL58796, and PAL58797. All these PAL specimens were collected by Vincenzo Tineo at Mondello, as indicated in the protologue [22] (p. 280). PAL58796 and PAL587967 includes a note in Tineo’s handwriting “*Salicornia fruticulosa Nob.* [nobis]” and the dates of collection (“*7bre* [settembre = September] *1827*” for PAL58796 and “*7bre 1826*” for PAL58797). Since the label of PAL58780 does not include the collection date, we cannot be sure that the plant was collected before 1827 (year of the original publication) and, therefore, we prefer to exclude it from the lectotypification. Among the other two specimens (which are clearly part of different gatherings), it is important to note that PAL58796 includes two parts of plants which are referrable to different species, i.e., *Salicornia perennis* (plant on the left, creeping and rooting at nodes and with its few terminal spikes being very short, up to 0.5 cm) and *S. fruticosa* (plant on the right, with many long spikes up to 4 cm), whereas PAL58797 cannot be identified since fertile branches are lacking [6,27]. Therefore, we here designated PAL58796 (plant on the right) as the lectotype of *Salicornia fruticulosa*.

#### 3.1.7. *Salicornia glauca*

Delile [36] (p. 49) published *Salicornia glauca* Del., citing “Salicornia virginica *Forskal.*” after his own new name. Delile described the *S. glauca* in order to correct Forsskal’s misidentification of the Linnaean *S. virginica* (see ‘Section 3.1.16 *Salicornia virginica*’). The abbreviation “As.” was also cited; this was taken from Forsskål’s explicatory notes [page “L” and LIX (Roman page numbers) on the first part of Forsskal’s *Flora Aegyptiaco-Arabica*] and it is not part of the name; it refers to the provenance and status of the species: “As. = Alexandriae spontaneae”. Based on the discussion in ‘Section 3.1.16 *Salicornia virginica*’ and on the ruling of ICN Art. 41.7 Note 3, Delile’s name is to be considered new to science, and *S. glauca* should replace Forsskål’s misidentified Linnaean name. However, *S. glauca* is an illegitimate and later homonym (Art. 53.1 of ICN [4]) of the identical name published the year before by Stokes [37] (p. 8), who discussed if his species *S. glauca* could be identical with *S. arabica* L. Note that *Salicornia arabica* is a name that was recently proposed for rejection [9]. Note also that the name of a new but previously misidentified species should be typified with original material relating to the new name, not automatically with material related to Forsskål’s misapplied name (ICN Art. 41.7 Note 3).

According to the HUH-Index of Botanists [38], Delile’s Egyptian collections (period 1798–1801) are preserved in several herbaria. We traced three specimens of *Salicornia glauca*, one at LINN-HS (no. 20-13, https://linnean-online.org/29388/#?s=0&cv=0&z=0.0657%2C0.3685%2C0.6281%2C0.7607, accessed 22 June 2024), one at P [barcodes P04918422 (specimen to the left on the sheet; image available at http://mediaphoto.mnhn.fr/media/1441381909236kOTJBPm8nnr55Zwf, accessed 22 June 2024), and one at P05234345 (image at http://mediaphoto.mnhn.fr/media/1441396286253vyQsUv9BFK48R0xt, accessed 22 June 2024). These samples were all collected by A.F. Delile in Egypt (as reported on the original labels). These specimens were clearly part of the material used by Delile to describe *Salicornia glauca*. According to the current concept in Salicornioideae [2,11], these specimens belong to the genus *Arthrocaulon*; this is based on the cymes being free and protruding. However, only the LINN-HS specimen is identifiable with certainty due to the presence of visible seeds that are glabrous, black, and shiny, which are diagnostic features of *Arthrocaulon*. Using species rank and based on the diagnostic features indicated in Ramírez et al. [3], LINN-HS20-13 can be identified as *A. meridionale* (spikes 5–6 cm long vs. up to 4 in *A. macrostachyum*). The occurrence of *A. meridionale* in Egypt is also congruent with the chorology of the species (south Mediterranean basin). We here designate the specimen LINN-HS20-13 as the lectotype of the name *Salicornia glauca* Delile since it appears to be much better preserved and richer in flowers than the two specimens at P. The features that can be seen on LINN-HS20-13 are important in the identification of the *Salicornia* species [6,27].

*Arthrocnemum glaucum* Ung.-Sternb., *nom. illeg.* (basionym *Salcornia glauca* Delile), was designated as being the type of *Arthrocnemum* in the enumeration of generic names in current use by Greuter et al. [39] (p. 86, see also the online version of the enumeration [40]). The designation is in agreement with the requirements in ICN Art. 7.11. According to ICN Art. 10.1, the type of the name of a genus is the type of the name of a species, and the lectotypification of *A. glaucum* and the identity of the type is therefore relevant. Greuter et al. [39] cites the name of *Arthrocnemum glaucum* Ung.-Sternb. without a basionym, as does IPNI [41], but the name is frequently cited in international databases as *Arthrocnemum glaucum* (Moq.) Ung.-Sternb., for example in POWO [42] and GBIF [43]. *Arthrocnemum glaucum* (Moq.) Ung.-Sternb. must be based on *Arthocnemum fruticosum* var. *glaucum* Moq. [30] (p. 112), published in 1840, where the informal name “Salicornia glauca plerumque Auct.” is cited as coming from Egypt and Syria without specified localities, collectors, or collections. However, according to the information published when the new combination *Arthrocnemum glaucum* was published by Ungern-Sternberg [44] (p. 283), its correct name is *Arthrocnemum glaucum* (Delile) Ung.-Sternb., basionym: *Salicornia glauca* Delile. Therefore, it must be typified with Delile’s original material of this species. It must, as shown above in our identification of Delile’s original material, be a heterotypic synonym of the much younger but still legitimate *Arthrocaulon meridionale*.

However, as mentioned in the general note in the introduction to this paper about our acceptance of the name *Arthrocaulon*, Pfeiffer’s lectotypification of *Arthocnemum* [8] (p. 279) with *Salicornia fruticosa* (L.) L. must stand. Pfeiffer’s typification is acceptable according to Art. 7.11, Ex. 17, and it has the priority of designation as the first lectotypification of a previously untypified name, which is to be followed according to ICN Art. 9.19. Standley [45] (p. 81) lectotypified *Arthrocnemum*, with “Arthrocnemum fruticosum Moq.” also cited as the accepted type of *Arthrocnemum* Moq. by the Missouri Botanical Garden’s database TROPICOS [46], but Standley’s typifications are rejected as using a mechanical method of type selection (ICN Art. 10.7), as is the report on mechanical methods for lectotypification by McNeill et al. [47] (p. 1447).

#### 3.1.8. *Salicornia lignosa*

Woods [48] (p. 31), in discussing Smith’s *Salicornia radicans* [25], proposed a new species (*S. lignosa* J. Woods) from Hailing Island (Hampshire, UK) that, according to the author, “somewhat resembles *S. radicans* in its diffuse mode of growth”. The diagnostic characteristics distinguishing between *S. lignosa* and *S. radicans* would be the “thickness and very firm structure of the lower part of the stem” (vs. “least solid stem”). Moreover, Woods [48] (p. 31) compared *S. lignosa* with *S. fruticosa*. These differ in terms of the length of the spike [“one inch or a little more long, about one-sixth this width” (*s. lignosa*) vs. “relatively longer” (*S. fruticosa*)].

As stated in the HUH-Index of Botanists [49], Wood’s herbarium and types are deposited at the herbaria BM, CGE, E, FI, K, MANCH, MNW, OXF, SLBI, and SWA. Unfortunately, no specimen of *Salicornia* was found to have been collected by Woods in these herabrai. Lacking original material, a neotypification was required (Art. 9.8 of ICN [4]). Among the relevant specimens found, seven [three at CGE (barcodes CGE00070856, CGE00070861, and CGE00070862), four at MANCH (no barcode; collections: Herb. P. M. Hall. no. 994, Herb. C. Bailey nos. 2510 and 2514, Herb. C. E. Salmon *s.n.*)] were collected in Hailing Island, i.e., the type locality of *Salicornia lignosa*. The specimens at MANCH are clearly annual and, therefore, cannot be referred to as Woods’ *Salicornia lignosa*, which is a woody perennial. Among the CGE specimens, CGE00070862 (R.S. Standen 1419) perfectly matches the diagnosis of *S. lignosa* and it is here designated as the neotype. This specimen is identifiable as *S. perennis* according to the current concept [2,11]. This is mainly for the habitus (creeping) and the characters of the terminal spikes, which are opposite and short, growing up to 2.0 cm.

#### 3.1.9. *Salicornia macrostachya*

**Figure 1:**
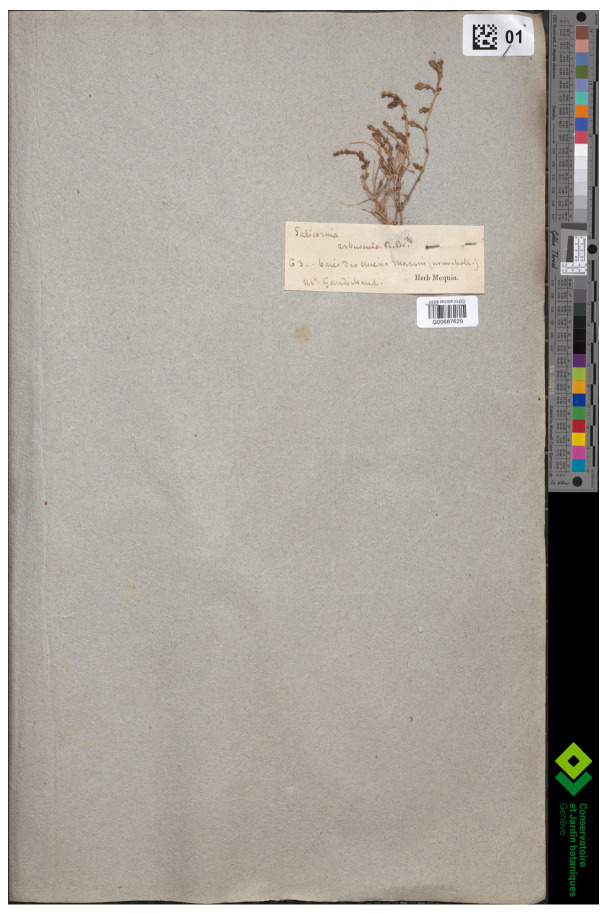
Specimen G00687629 of *Salicornia arbuscula* collected by C. *Gaudichaud*-Beaupré.

*Salicornia macrostachya* Moric. was published by Moricand [50] (p. 2), who provided a diagnosis (“S. [Salicornia] caule fruticoso, ramis ascendentibus, articulis superioribus vix crassitie longioribus, spicis crassis subclavato-cylindraceis, sessilibus”) and its provenance (“Hab. secus viam quae ad portum *Malamocco* ducit …”, where “Malomocco” was the first settlement on the Lido of Venice barrier island of the Lagoon of Venice, Veneto region, north-eastern Italy). Further, a name from the Candolle herbarium was cited, i.e., “S. [Salicornia] arbuscula *De C. herb.* non *Brown. nov. holl.*”. The citation of a specimen included in the Candolle herbarium (note that Candolle “S. arbuscula” is not *S. arbuscula*, published by Brown [51] (p. 411), currently accepted as *Tecticornia arbuscula* (R.Br.) K.A. Sheph. and Paul [52]), must refer to a syntype according to Art. 9.6 of ICN [4]. (Candolle’s collections are currently mainly preserved at G and G-DC [53].) The name “*Salicornia arbuscula* DC.” was published by Moquin-Tandon [30] (p. 112) and [54] (p. 151) in synonymy with *Arthrocnemum fruticosum* var. *macrostachyum* (Moric.) Moq. (as “S. arbuscula DC! *herb* (v.v.)”). However, being published in synonymy, Moquin-Tandon’s *Salicornia arbuscula* is not validly published according to Art. 36.1b of ICN [4]. The exclamation mark indicates that Moquin-Tandon had seen a specimen with that name, and “v.v.” (*vidi vivo*) suggested that he had also seen living plants. We found one specimen at G (G00687629; Figure 1), bearing one plant and the following label: “*Salicornia arbuscula R. Br.*|*63. baies des Chiens Marim (nouv. holl.)*|*M^r^ Gaudichaud*|Herb. Moquin”. It thus came from Moquin-Tandon’s herbarium but, being collected from Australia [‘*nouv. holl.’*] by Charles Gaudichaud-Beaupré, it must represent Robert Brown’s *S. arbuscula* and not “S. arbuscula DC! *herb”.* In fact, G00687629 cannot be mistaken for *A. macrostachyum* (Moric.) Piirainen and G. Kadereit because of its habit [a tiny and probably decumbent subshrub (chamaephyte) vs. robust and erect shrub (phanerophyte)]. It seems that this specimen is the real *S. arbuscula* R.Br. (currently *Tecticornia arbuscula*), an Australian endemic species [50]. It would also seem that Moricand may have referred to another specimen in Candolle’s herbarium than G00687629, because Gaudichaud returned from his circumnavigation in 1820, the year of the publication of Moricand’s flora [50]. Note that the name “Salicornia arbuscula” was also published in synonymy of *Salicornia macrostachya* Moric. by Steudel [55] (p. 493). Whatever the identity of the specimen “S. [Salicornia] arbuscula *De C. herb.*”, Moricand [50] (p. 2) validly and legitimately published the name of the species based on more than one collection and under the name *Salicornia macrostachya*, which was new to science at the time of publication and not a *nomen novum* or validation of *S. arbuscula* sensu Candolle.

Piirainen et al. [2] (p. 123) indicated that the specimen G00177362 was the holotype of *S. macrostachya*. However, Moricand [50] (p. 2) did not specify any holotype (see Art. 9.1 of the ICN [4] and the considerations by McNeill [56]) and cited a syntype; lectotypification was therefore necessary. We examined G00177362 and verified that M. Ph. Moricand (the grandson of M.-E. Moricand) gave it to the Herbarium G in 1908 (as reported in the printed label on the top of the sheet). The handwritten label (top-right of the sheet) states “*Salicornia macrostachya Moric.*|*ex ipso!*|*Des environs de Venice malamocco 7^bre^* [September]”. This specimen was collected by M.-E. Moricand, as clearly indicated in the printed label (“Les étiquettes non signees, se rapportant à des plantes d’Europe, proviennent des herborisations personnelles de Moricand” = “The unsigned labels, relating to European plants, come from Moricand’s personal collections”). Although the collecting locality cited in the protologue is mentioned on the label, the information about G00177362 does not include the year of collection (only the month is reported). Therefore, we cannot be sure that it is an ante-1820 addition to Moricand’s collection and may not part of the original material of the name *Salicornia macrostachya*. Fortunately, we traced another specimen (G00687638) with a plant collected in the “*environs de Venice*” by Moricand in 1818 (with that year and also “*Salicornia macrostachya N.* [Nobis]” written on the specimen). Thus, the specimen in G-DC, G00687638, is certainty part of the original material.

All things considered, the typification by Piirainen et al. [2] (p. 123) is not correct, their search for original material was not adequate, and Art. 9.10 of ICN [10] (correction of the term holotype to lectotype) cannot be applied. G00687629 is not identifiable with *A. macrostachyum* (Moric.), published by Piirainen and G. Kadereit [possibly the Australian endemic *S. arbuscula* R.Br. (currently known as *Tecticornia arbuscula*)]. Therefore, G00687638 is the only extant material clearly part of the original material and useful for lectotypification, and it is here designated as the lectotype of *Salicornia macrostachya*. This lectotype morphologically matches Moricand’s description, being a fruticose and fleshy plant with ascending branches and sessile and cylindrical inflorescences. Concerning the current identity of G00687638, we note that Moricand’s species is currently accepted under the genus *Arthrocaulon* Piirainen and G. Kadereit as *A. macrostachyum* [2] (p. 123).

#### 3.1.10. *Salicornia macrostachya* Varieties by Michele Tenore

As mentioned in ‘Section 3.1.3 *Salicornia fruticosa* varieties by Michele Tenore’, Tenore [31] (p. 5), in volume IV of his *Flora Napolitana*, recognized *Salicornia macrostachya* as a variety of *S. fruticosa*. As regards habitat and provenance, he reported “Ibidem”, referring to the localities cited just above under *S. fruticosa* (sensu stricto), i.e., “In inundatis salsis. *Fusaro*, *Maremorto*, *Lago salso*”. One year later, Tenore [24] (p. 582) accepted *S. macrostachya* at the species rank and classified it into two varieties (described as new), i.e., var. *virescens* Ten. (diagnosis: “elata, caulis valde lignosis (digitalis crassitici bipedalis altitudinis) ramisque strictis. Planta laete virens fere arborescens nunquam gregarie crescens”) and var. *glaucescens* Ten. (diagnosis: “patula depressa, caulibus fruticulosis (1-3-linearis crassitici pedalis altitudinis), ramisque divaricatis prostratis. Planta viridi-glauca suffruticosa passim radicans”); no further data were given by Tenore [24] (p. 582) for these varieties, and he made no direct or indirect references to the varieties descibed in 1827 under *S. fruticosa*. We traced two specimens at NAP [32] that bore original labels annotated with “*Salicornia macrostachya virescens ...*|*Miseno*” (barcode NAP0000047) and “*Salicornia macrostachya glaucescens ...*” (barcode NAP0000048); NAP0000048 also bore a second label, reporting “*Salicornia fruticosa*|*Mare morto*”. Note that *Miseno* refers to a coastal lake included in the territory of Bacoli Municipality (Province of Naples, Campania region, southern Italy) and that *Mare morto* is a local name for this lake (E. Del Guacchio pers. comm.). These localities match those reported for *S. fruticosa* in volume IV of Tenore’s *Flora Napolitana*, as indicated above. Unfortunately, no date of collection was reported in these two NAP specimens and, therefore, we cannot be sure that they are ante-1831 collections. As a consequence, we prefer to avoid their use as lectotypes (Art. 9.3 and 9.4 of ICN [4]). Since no further specimen of the original material was found, a neotypification was required according to Art. 9.8 of ICN [4] and we designated NAP0000047 as the neotype of *S. macrostachya* var. *virescens* and NAP0000048 as the neotype of *S. macrostachya* var. *glaucescens*. Both the neotypes were identifiable as *Arthrocaulon macrostachyum* (we observed undivided hollows in the segments where the flowers had fallen) according to the current concept [2,11]. Note that Tenore [31] indicated “passim radicans” (= sometimes rooting) for var. *glaucescens* (this is a character which could link his concept to *Salicornia perennis*); however, the neotype does not appear to creep and, therefore, it possible that Tenore observed (but did not collect) the first internodes near the main root.

#### 3.1.11. *Salicornia perennis*

Miller [57] (“Salicornia no. 2 (‘perenne’)” and in the corrigenda “Salicornia 2, lege (Perennis)”) described *S. perennis* Mill. from plants growing in Sheepy Island. Guilló et al. [58] (p. 333) proposed a neotype for Miller’s name on a sheet deposited at K (K000450665). The material on K000450665 is represented by two sterile plants and, according to the current concept [6,27], sexual character is almost always essential for the identification of taxa of *Salicornia*. Therefore, an epitypification might be desirable (Art. 9.9 of ICN [4]). However, the neotype is a suffruticose plant, with green, diffuse stems; rooting at the nodes, with numerous short and creeping sterile branches (even underground); short ascending stems, which are slightly lignified; and fine, slightly thickened segments. These morphological features allow us to identify the plant on K000450665 as *Salicornia perennis*.

#### 3.1.12. *Salicornia radicans*

Smith [25] (unpaginated text opposite Tab. 1691) proposed his *Salicornia radicans* Sm. with a short diagnosis (“Stem woody; procumbent and taking root at the base. Joints compressed, notched; interstices nearly cylindrical. Spikes oblong. Style deeply divided. Stamens two”). An illustration (Table 1691) was also published, and it is part of the original material. However, Smith [25] also reported two collections (syntypes according to Art. 9.6 of ICN [4]). One was collected by the reverend C. Sutton (in September 1798) from “sea coast at Holm, Norfolk”; the second one was taken by Mr. W. Borrer (no date indicated) from the “harbour at Shoreham, Sussex”. The locality of Weymouth (Dorset, UK) is also reported. We traced the two syntypes at LINN-HS (where Smith’s herbarium and types are preserved [59]). One sheet (LINN-HS20-6) bears one plant and the following annotation (on the bottom-left corner of the sheet): “*Coast of Sussex. M^r^ Borrer. 1805*”. The other sheet bears two plants (barcoded as LINN-HS20-5-1, LINN-HS20-5-2) and is annotated with the numbers “*1*” and “*2*”. This links the specimens to the notes on the bottom-left corner of the sheet: “*Holm Norfolk. Rev. C. Sutton (D.D.) 1798*” (no. 1, barcode LINN-HS20-5-1) and “*Norfolk coast. M^r^ Borrer. 1806*” (no. 2, barcode LINN-HS20-5-2). According to the protologue, of those displayed on this sheet, only LINN-HS20-5-1 is a syntype, whereas LINN-HS20-5-2 cannot be since, as reported in the protologue [25], the specimen cited in the protologue was collected by M^r^ Borrer in Norfolk County, not in Sussex County. Both syntypes (LINN-HS20-6) and (LINN-HS20-5-1) are identifiable as *S. perennis* according to the current concept [2,11]. We here designate LINN-HS20-6 as the lectotype for *S. radicans* since the other syntype (Suttons’ collection) is represented by a small fragment of the terminal part of the inflorescence, whereas Borrer’s specimen is complete. Although Smith’s illustration (Table 1691) is part of the original material, ICN [4] states that when syntypes occur, the lectotype must be chosen among them (Art. 9.12 and Ex. 12 of ICN [4]). In the glossary of the ICN [4] (p. 202) a “syntype” is defined as including “Any specimen cited in the protologue when there is no holotype, …”.

#### 3.1.13. *Salicornia radicans* var. *caespitosa*

Rouy [34] (p. 60) described a variety of *Salicornia radicans*, *S. radicans* var. *caespitosa* Rouy, which was characterized by “Plantae relativ courte, en touffe ou buissonnante; épis gros, cylindriques, faibl fructifères ou stériles”; the provenance was also reported (“*Grande-Bretagne: Péninsule ibérique*”).

Unfortunately, no specimen that would be useful for the lectotypification purpose was traced at LY, where Rouy’s herbarium and types were preserved (M. Thiebaut pers. comm.). Therefore, a neotypification would be desirable (Art. 9.8 of ICN [4]). However, Rouy [34] (p. 60) did not specify any locality, providing a wide distribution area (Great Britain and the Iberian Peninsula). It is therefore difficult to select a collection made at a reasonably narrowly defined *locus classicus* as the neotype. The original description is quite vague but is based on these characteristics: “Plantae relativ courte, en touffe ou buissonnante” (= plant relatively short in tufts or shrub). Thus, Rouy’s variety falls within the circumscription of *Salicornia perennis* according to the current concepts [2,6]. Concluding, we here refrain from the typification of *Salicornia radicans* var. *caespitosa* but propose it to be placed in the synonymy of *S. perennis.*

#### 3.1.14. *Salicornia sarmentosa*

Duval-Jouve [60] (p. 174) described *S. sarmentosa* Duval-Jouve, providing a detailed description. Information on habitat and provenance (“Bords de étangs et des marais salants, dans les lienx très-humides et inondés hiver ... Aigues Mortes ... à Carnon ... entre Palavas et Maguelonne ... à Vic; à Frontigna; aux Onglous ... Saint Vaast (Manche), d’où M. le docteur Lebel”) was also provided, and it was suggested (“an”) that it was close to but not a synonym of Tenore’s *Salicornia fruticosa* var. *humilis* (the latter would have made S. sarmentosa an illegitimate renaming). We found sixteen relevant specimens at MPU (where Duval-Jouve’s collection is mainly preserved) and P (where there are duplicates of the MPU specimens) [61]. The nine specimens found at MPU were MPU227866, MPU259991, MPU1316700, MPU1316694, MPU1323504, MPU1323505, MPU1323510, MPU1323511, and MPU1323513, and the collections were made at Vic (4), Maguelonne (2), and Palavas (1), which are localities cited in the protologue. However, six out of these nine specimens were collected after the date of publication (1869, 1877, and 1887), whereas the other ones were syntypes (Art. 9.6 of ICN [4]) and original material for *Salicornia sarmentosa* (years of collection: 1862 and 1868). Seven specimens were found at P (barcodes P06739611, P05158076, P05235503, P00724221, P00724223, P00724224, and P00724226) but they were all collected after 1868. Therefore, the P specimens cannot be considered to be part of the original material for *Salicornia sarmentosa*. Among the three MPU specimens that are original material, one was collected at Maguelonne in 1842 (MPU1316694), with the other two (MPU1316700 and MPU1323511) collected at Vic in 20 September 1868. We here designate the MPU1316700 specimen, which is part of “HERBIER DE LA FLORE DEL MONTPELLIER PAR Lt. [Loret] et B [Borrandon]” (as indicated in the original label on the bottom-right corner of the sheet), as the lectotype of the name *Salicornia sarmentosa*. According to De La Fuente et al. [33], its type cannot be identified. According to these authors, MPU1316700 can be referred to either *Saronornia perennis* (Mill.) A.J. Scott. (currently *Salicornia perennis*) or *Sarcocornia alpinii* (Lag.) Rivas Mart. (*Salicornia alpinii s.s.* according to [2]), but seeds are lacking. Further investigations (field surveys) are necessary to reach a taxonomic conclusion about the identity of the name. As a consequence, we prefer to avoid synonymizing these names, which are presented as separate (see ‘Section 3.2 Taxonomic Treatment’).

#### 3.1.15. *Salicornia sempervirens*

Steudel [62] (p. 714) published the name “*Salicornia sempervirens Sauvag.*” As a synonym of the validly published *S. fruticosa*. According to Art. 36.1a of ICN [4], Steudel’s name is not validly published.

#### 3.1.16. *Salicornia virginica*

A plant called *Salicornia virginica* was first validly described and named by Linnaeus [63] (p. 4) in the 1st Edition of *Species Plantarum*. *Salicornia virginica* L. is currently considered the correct name for a species native to N. America (S. Alaska and coastal areas on the eastern and western sides of Canada and USA), N. Mexico, and the Caribbean [64]. However, note that Ball [27] (p. 384) accepted *S. depressa* Standl. as the name for this American plant, with *S. virginica* as a possible synonym, and that Piirainen et al. [2] (p. 124) indicated *S. depressa* as a doubtful heterotypic synonym of *S. virginica* L., whereas POWO [64] and Southeastern Flora [65] reported *S. depressa* as a sure synonym of the Linnaean plant. Information about the previous lectotypification of *S. virginica* is shown below.

In *Flora Aegyptiaco-Arabica* [66], brought to the press by an anonymous editor 12 years after Forsskål died on the expedition to Egypt and Arabia, the name *S. virginica* is listed in two places in the text with reference to a plant from Egypt. This includes Forsskål [66] (p. LIX, No. 3): “[Salicornia] virginica, farinosa.—As.” Here, the typography indicates that “*virginica*” is an epithet, and that “farinosa” is a descriptive term, and “As” is an abbreviation for the locality and status, i.e., “Alexandria spontanea”]. This also includes Forsskål [66] (p. 2, No. 2): “SALICORNIA virginica. Arab. Chraesi.” This gives the Latin name of the species and the vernacular name in Arabic, followed immediately by a description of the species. Forsskål’s *S. virginica* was considered a species new to science by Christensen [67] (p. 10) and several subsequent authors. If this interpretation is correct, then “*S. virginica* Forssk.” is a later homonym of the Linnaean *S. virginica* and, hence, Forsskål’s name would be illegitimate under Art. 53.1 of ICN. This is also how POWO [68] previously interpreted the situation, accepting *Salicornia virginica* Forssk. as an illegitimate heterotypic synonym of *Arthrocaulon macrostachyum*. Piirainen et al. [2] (p. 123) cited Forsskål’s name of “*Salicornia virginica* Forssk., Fl. Aegypt.-Arab.: 2. 1775, non L. 1753” as a heterotypic synonym of *Arthrocaulon macrostachyum*. However, *S. virginica* sensu Forssk. is a misidentification in the sense of the Shenzhen Code [4] (p. 124), and it is, according to the Recommendation 50D of the ICN, to be indicated by the words “auct. non”, followed by the name(s) of the original author(s) and the bibliographic reference of the misidentification. This was originally demonstrated by Hepper and Friis [69] (p. 101), who cited Forsskål’s name as “*Salicornia virginica* sensu Forssk. 1775: 2 (LIX no. 3; Cent I No 2), non L. (1753)”, which is different from the format of ICN but has the same meaning.

In order to document this point, we have re-examined the conclusion by Hepper and Friis [69] (p. 101), not only regarding Forsskål’s use of *S. virginica*, but also other parallel cases of Linnaean names for American plants listed as accepted names in *Flora Aegyptiaco-Arabica*. We know that Forsskål, on the expedition to Egypt and Arabia, worked with a very limited number of books (Hepper and Friis [69] (p. 25)). Moreover, Hepper and Friis ([69] (pp. 25–26)) observed that “he expected to find American and Asian plants in Egypt and Arabia … He seems often to have accepted an identification if the plant matched a description in the Linnaean works he had with him, no matter where the species was [originally] described from. He used the botanical books of Linnaeus as a world flora.”. This conclusion is documented by statements made by Forsskål himself on the journey, for example in a letter to Linnaeus from Yemen: “Here I have found a lot of American, Indian, and new plants …”. A total of 188 cases of Forsskål’s misidentifications of Linnaean names can be found among the names listed by Hepper and Friis [69]. As a further argument for considering Forsskål’s identification of his Egyptian plant with *S. virginica*. L. as a misidentification, it should be noted that Forsskål’s [66] (p. 2) description of *S. virginica* included part of Linnaeus’s diagnostic phrase for *S. virginica* L. almost *verbatim* [“*Articulis* … apice compressiusculi, emarginato bifidi” (in *Flora Aegyptiaco-Arabica*) vs. “articulis apice compressis emarginatis bifidis” (in *Species Plantarum*)].

**Figure 2:**
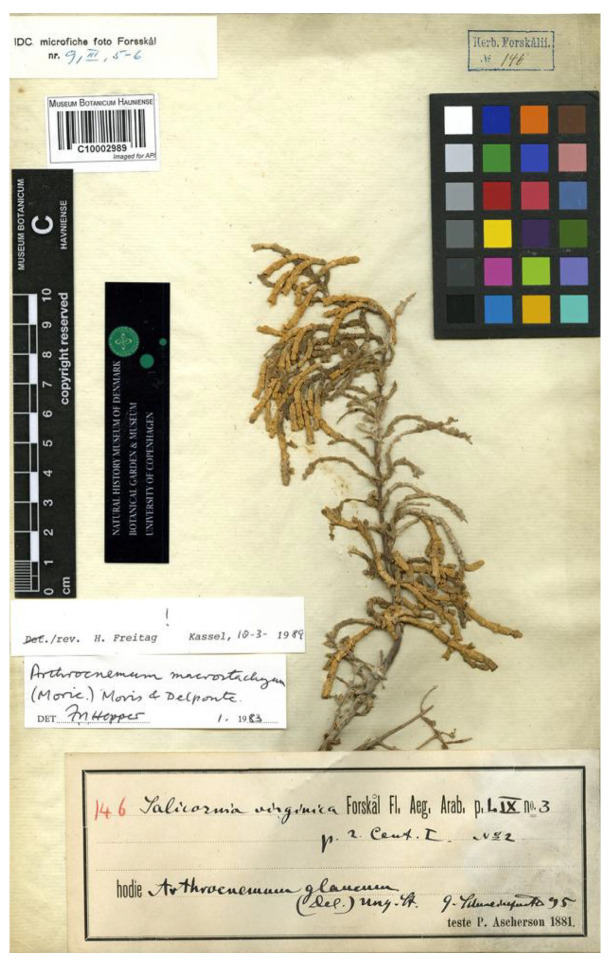
The best preserved and most complete of three sheets collected at Alexandria, Egypt, and identified by Forsskål (C!) as *S. virginica* auct. non. L.: Forssk., Fl. aegypt.-arab.: p. LIX, No. 3, and p. 2, No. 2 (1775). Reproduced with permission from the Natural History Museum of Denmark.

However, does Forsskål’s plant in fact differ from the Linnaean *S. virginica* and, if so, how, and what is its identity? We studied the original material in Forsskål’s herbarium at C [70], which contains three specimens of *S. virginica* sensu Forssk., which are also referred to here with their old collection numbers used in the work of Hepper and Friis [69] (p. 101), links to images, and notes about the presence and length of inflorescences (Figure 2):

C10002945 (old collection number 169, image on https://plants.jstor.org/stable/viewer/10.5555/al.ap.specimen.c10002945, accessed 22 June 2024; with a few intact inflorescences, ca. 2.2 cm long).

C10002989 (old collection number 146, image on https://plants.jstor.org/stable/viewer/10.5555/al.ap.specimen.c10002989, accessed 22 June 2024; with a few intact inflorescences, ca. 2.3 cm long).

C10002990 (old collection number 174, image on https://plants.jstor.org/stable/viewer/10.5555/al.ap.specimen.c10002990, accessed 22 June 2024, with no intact inflorescences).

These three specimens were all collected by Forsskål near Alexandria in Egypt and are part of the material used by him to write the description of his *S. virginica*.

Although Forsskål’s plants clearly belong to the genus *Arthrocaulon*, it is, as for Bertoloni’s specimens discussed above in ‘Section 3.1.2 *Salicornia fruticosa* var. *β by Antonio Bertoloni*’, not a simple issue of identifying them as either *Arthrocaulon macrostachyum* or *A. meridionale.* The quantitative diagnostic features given by Ramírez et al. [7] partially overlap or are very near to each other. The best characteristic for this seems to be inflorescence length, but Forsskål’s plants have very few intact and apparently unripe inflorescences, as outlined above. In the lists of Ramírez et al. [3] with diagnostic features of *A. macrostachyum*, the inflorescence length is given in cm as “(2.5) 2.9 (4)”, and for *A. meridionale* it is given as “(2.5) 3.8 (5.5)”. As such, the preserved spikes on Forsskål’s plants are shorter than the intervals given for both species, but perhaps verge towards *A. macrostachyum*. The real diagnostic feature according to Ramírez et al. [3] is that *A. macrostachyum* is diploid and *A. meridionale* is tetraploid. Not being able to test the ploidy of Forsskål’s plants, as with the identification of Bertolini’s collections in ‘Section 3.1.2 *Salicornia fruticosa* var. *β*’, one may have to rely on chorology. From this, Forsskål’s plants could be identifiable as *A. meridionale*, a species which according to Ramírez et al. is distributed throughout North Africa [3]. However, Ramírez et al. [3] do not cite identified specimens from Egypt, where *A. macrostachyum* is widespread according to Boulos [71] (p. 108), nor from the coasts of the Red Sea and the Gulf of Aden, from where all material was identified as *A. macrostachyum* before a distinction emerged between this and *A. meridionale* (see below). In ‘.2. Taxonomic treatment’, we have tentatively referred Forsskål’s Egyptian plants in the synonymy of either *A. macrostachyum* or *A. meridionale*.

On the contrary to the situation with the plants in Egypt, *Salicornia virginica* of Linnaeus [63] (p. 4) is an annual species [27] (p. 384), the lectotype of which is a Clayton specimen, no. 572/667, BM000051639, (image on https://data.nhm.ac.uk/dataset/collection-specimens/resource/05ff2255-c38a-40c9-b657-4ccb55ab2feb/record/4748367, and https://plants.jstor.org/stable/viewer/10.5555/al.ap.specimen.bm000051639; both images accessed on 22 June 2024). The lectotype was selected by Fernald and Schubert [72] (p. 163). Piirainen et al. [2] (pp. 113, 124) placed it in the *Salicornia* subgen. *Salicornia*, and it is accepted as the correct name of a North American species by POWO [64].

It should finally be noted that the wide distribution of *A. meridionale*, indicated here and outlined in [3], is not currently accepted in POWO [73], which only accepts the distribution on Sicily. It should also be noted that *Arthrocnemum*/*Arthrocaulon macrostachyum* has a much wider distribution than that recorded by Ramírez et al. [3]. It reaches south through most parts of Egypt [71] (p. 108), along the Red Sea coast in Sudan [74] (p. 277), in Eritrea [75] (pp. 289–290), in Yemen [76] (p. 84), along the Gulf of Aden down to Socotra, and in the inland localities of southern Somalia [77] (p. 131). Moreover, POWO [78] indicates that in addition to the above distribution, *A. macrostachyum* is recorded in Saudi Arabia, Jordan, Oman, United Arab Emirates, Iran, and Pakistan. The identity of the plants from these areas was not discussed by Ramírez et al. [3], and more statements about the distribution of *Arthrocaulon macrostachyum* and *A. meridionale* must be postponed until further studies have been conducted on the taxonomy of these two species and their distribution.

In conclusion, Forsskål did not consider his *S. virginica* to be a name for a hitherto unknown species; he thought it could be identified with the Linnaean *S. virginica* from North America. It should therefore be referred to as *Salsola virginica* auct., non L.: Forsskål, *Fl. Aegypt. Arab*.: LIX; 2 (1775). The many other cases where Forsskål identified his Middle Eastern and Arabian plants with names previously proposed by Linnaeus should be treated in the same way as such issues were resolved here for *S. virginica*.

### 3.2. Taxonomic Treatment

***Arthrocaulon macrostachyum*** (Moric.) Piirainen and G.Kadereit, Taxon 66(1): 123. 2017 ≡ *Salicornia macrostachya* Moric., Fl. Venet. [Moricand] 1: 2. 1820 ≡ *Arthrocnemum fruticosum* var. *macrostachyum* (Moric.) Moq., Chenop. Monogr. Enum.: 112. 1840 ≡ *Salicornia fruticosa* var. *pachystachya* W.J.D.Koch, Syn. Fl. Germ. Helv., ed. 2: 693. 1844, *nom. superfl. et illeg.* (Arts. 52.1 and 52.4 of ICN [4]) ≡ *Arthrocnemum macrostachyum* (Moric.) K. Koch, Hort. Dendrol.: 96. 1853.

**Figure 3:**
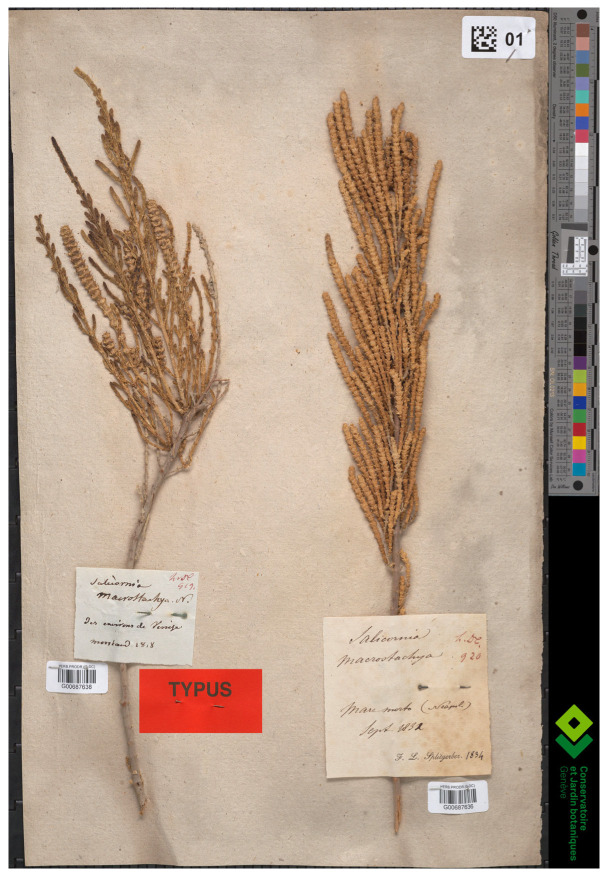
Lectotype of *Salicornia macrostachya* (G00687638!, plant to the left). ©: Conservatoire et Jardin botaniques de la Ville de Genève.

Lectotype (here designated):—ITALY. Des environs de Venice, 1818, *Moricand s.n.* (G-DC; G00687638! (left specimen) (Figure 3).

= *Salicornia macrostachya* L. var. *glaucescens* Ten., Syll. Pl. Fl. Neapol.: 582. 1831, *syn. nov*.

**Figure 4:**
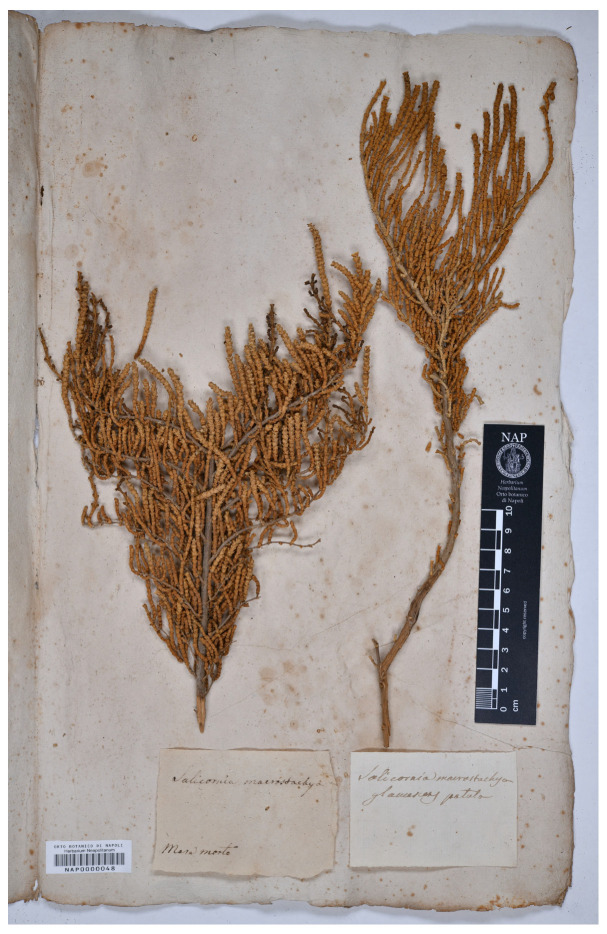
Neotype of *Salicornia macrostachya* var. *glaucescens* (NAP0000048!).

Neotype (designated here)—ITALY. Campania, *Mare morto*, *s.d.*, *M. Tenore s.n.* (NAP0000048; Figure 4).

= *Salicornia macrostachya* var. *virescens* Ten., Syll. Pl. Fl. Neapol.: 582. 1831, *syn. nov*.

**Figure 5:**
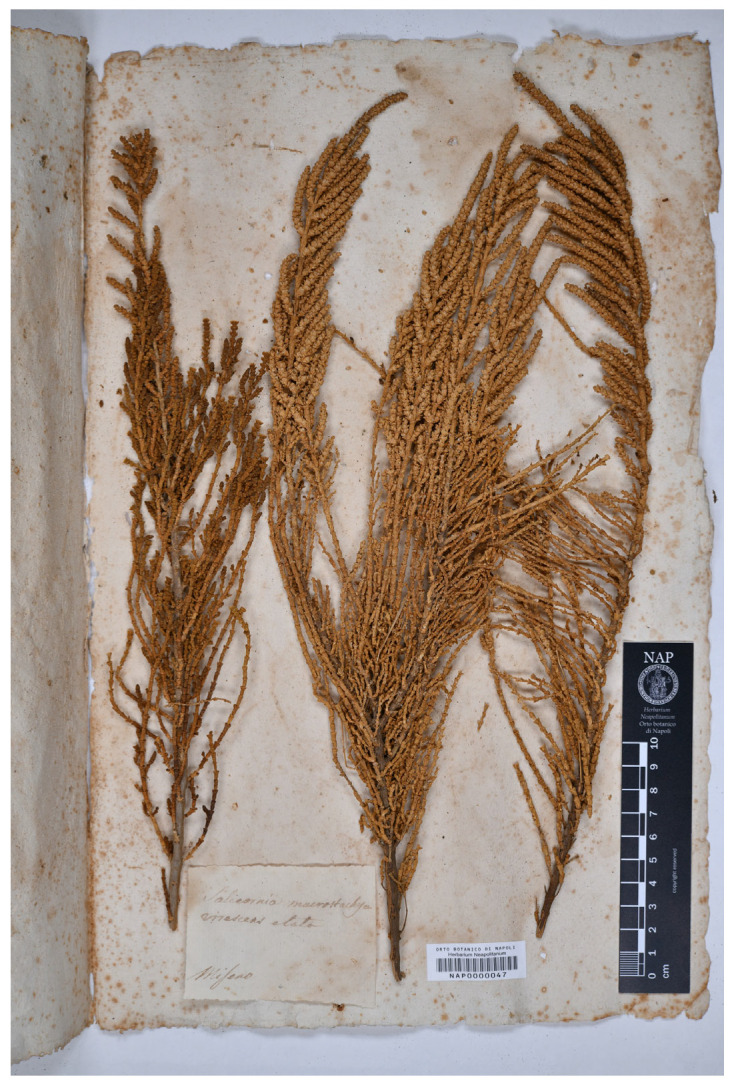
Neotype of *Salicornia macrostachya* var. *virescens* (NAP0000047!).

Neotype (designated here)—ITALY. Campania, Miseno, *s.d.*, *M. Tenore s.n.* (NAP0000047; Figure 5).

− *Salicornia arbuscula* Steud., Nomencl. Bot. [Steudel], ed. 2. 2: 493. 1841, *nom. inval. pro syn.* (Art. 36.1b of ICN [4]).
− *Salicornia virginica* auct. non L. (Sp. Pl. 1: 4. 1753): Forssk., Fl. Aegypt.-Arab.: LIX, 2. 1775 [tentative synonymy; either a synonym of *A. meridionale* or this name, due to doubt as to the identification of Forsskål’s material as either *A. macrostachyum* or *A. meridionale*].

*Other specimen seen*. Italy: Des environs de Venice, Malomocco, September, *Moricand s.n.* (G00177362!).

***Arthrocaulon meridionale*** [as ‘*meridionalis*’] Est.Ramírez, Rufo, Sánchez Mata, V. Fuente, Medit. Bot. 40(1): 34. 2019. ≡ *Arthrocnemum meridionale* (Est.Ramírez, Rufo, Sánchez Mata and Fuente) Fuente, Sánchez-Gavilán, Est.Ramírez, Rufo and Sánchez-Mata, in M.N. Grigore (ed.), *Handbook of Halophytes*. Springer, Cham: 1249. 2021, DOI: 10.1007/978-3-030-57635-6. [The new combination is accepted as validly published from the 2021 edition by IPNI, not the online preprint from 2020, and ascribed to the authors of the publication in which the new combination appears (Art. 46.5 and 46.6 of the ICN [4])].

Holotype (Ramírez et al. [3] (p. 34)):—ITALY, Sicily, between Trapani and Paceco. ‘Saline di Trapani e Paceco’ nature reserve, perennial halophytic communities close to Salina Chiusicella (Salicornietea fruticosae), 14 June 2017, *V. de la Fuente*, *N. Rodríguez and D. Sánchez-Mata* (MAF176512).

= *Salicornia glauca* Delile, Fl. Aegyp. Illustr. 1: 49. 1813, *syn. nov.*, *nom. illeg.* (Art. 53.1 of ICN [4]), non Stokes (1812: 8) ≡ *Arthrocnemum fruticosum* var. *glaucum* (Delile) Moq., Chenop. Monogr. Enum.: 112. 1840, *nom. illeg.* ≡ *Arthrocnemum glaucum* (Delile) Ung.-Sternb., Atti Congr. Int. Bot. Firenze 1874: 283. 1876, *nom. illeg.*

Lectotype (designated here):—EGYPT. *S.d.* (period 1798–1801), *A. R. Delile s.n.* (LINN-HS20-13!, image of lectotype available at https://linnean-online.org/29388/#?s=0&cv=0&z=0.1054%2C0.2356%2C0.6281%2C0.7607, accessed on 22 June 2024).

− *Salicornia virginica* auct. non L. (Sp. Pl. 1: 4. 1753): Forssk., Fl. Aegypt.-Arab.: LIX, 2. 1775 [tentative synonymy; either a synonym of *A. macrostachyum* or this name, due to doubt as to the identification of Forsskål’s material as either *A. macrostachyum* or *A. meridionale*].

***Salicornia fruticosa*** (L.) L., Sp. Pl. ed. 2 1: 5. 1762 ≡ *Salicornia herbacea* L. var. *fruticosa* L., Sp. Pl. 1: 3. 1753 ≡ *Arthrocnemum fruticosum* (L.) Moq., Chenop. Monogr. Enum.: 111. 1840 ≡ *Sarcocornia fruticosa* (L.) A.J.Scott, Bot. J. Linn. Soc. 75: 367. 1977.

**Figure 6:**
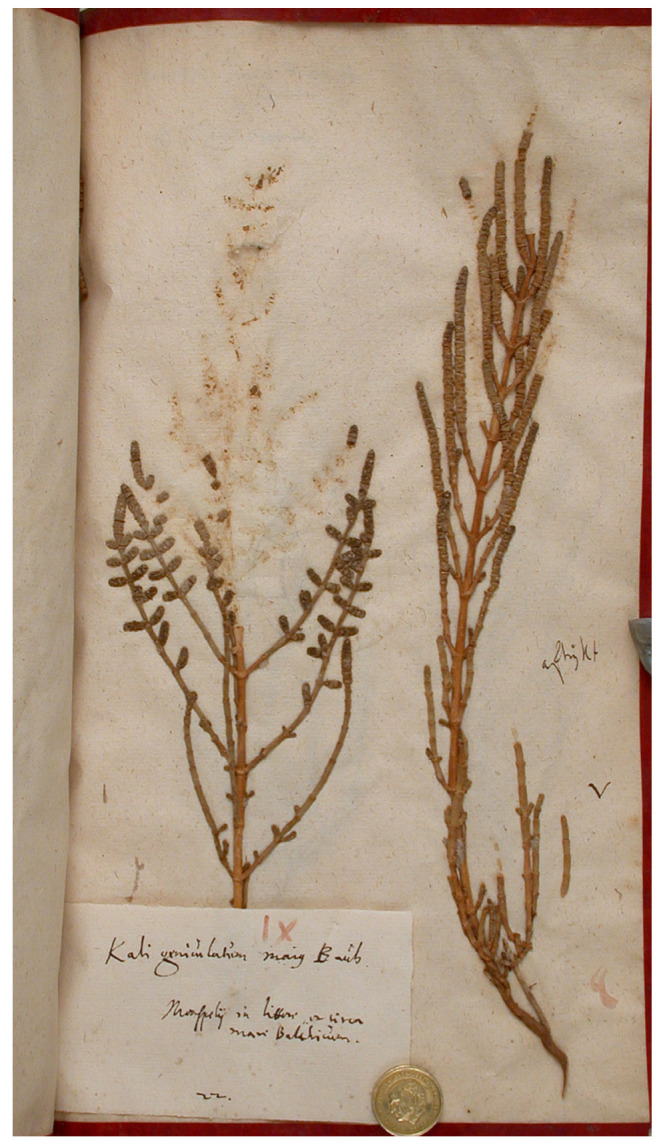
Lectotype of *Salicornia fruticosa* (Herb. Burser XVI(2): 22, UPS, plant to the right. Reproduced under the terms of the Creative Commons Attribution License [CC BY 4.0], Museum of Evolution, Uppsala University.

Lectotype (designated by Ball [79] (p. 807)):—EUROPE. Monspelii in littore, et circa mare Balticum. Sub Kali geniculatum maius Bauh., Herb. Burser XVI(2): 22 (UPS!, plant to the right; Figure 6).

= *Salicornia fruticulosa* Tin., Cat. Pl. Hort. Panorm.: 280. 1827.

Lectotype (designated here):—ITALY. Sicily, Mondello, September 1827, *V. Tineo s.n.* (PAL58796! [plant to the right], image of lectotype available at https://herbarium.unipa.it/zoomify/view_img.asp?ic=58796A new, accessed on 22 June 2024).

= *Salicornia fruticosa* L. var. *glaucescens* Ten., Syll. Pl. Fl. Neapol.: 582. 1831.

**Figure 7:**
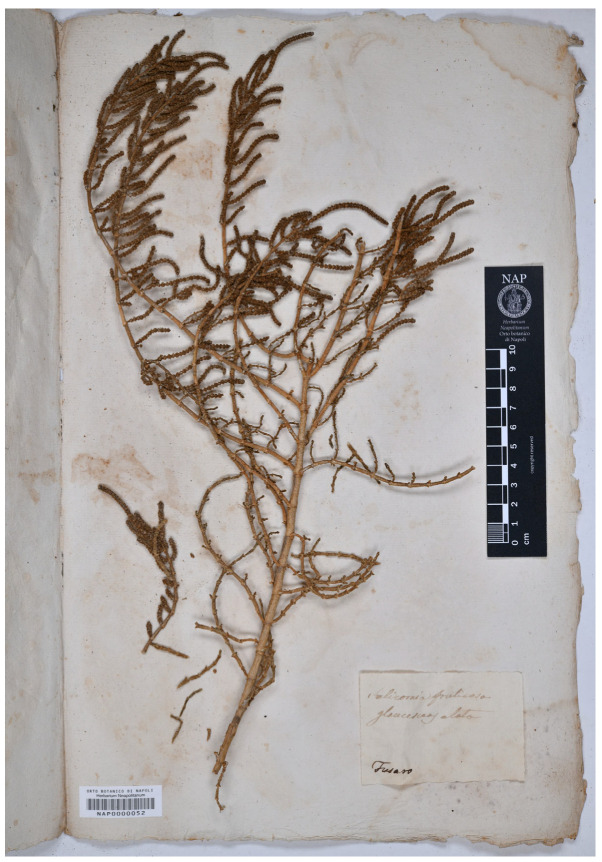
Neotype of *Salicornia fruticosa* var. *glaucescens* (NAP0000052!).

Neotype (designated here)—ITALY. Campania, Fusaro, *s.d.*, *M. Tenore s.n.* (NAP0000052; Figure 7).

= *Salicornia deserticola* A.Chev, Rev. Bot. Appl. Agric. Trop. 1934, xiv: 804. 1934.

Lectotype (designated by Piirainen [80] (p. 107)):―ALGERIA. Sud Algérien, Témacine prés Touggourt, terrains salés, 15 décembre 1931, *A. Chevalier 42063* (P00713535!, image available from http://science.mnhn.fr/institution/mnhn/collection/p/item/p00713535, accessed on 22 June 2024; isolectotypes: P00713536!, P01817707!, P01817708!, P01817709!).

***Salicornia perennis*** Mill., Gard. Dict., ed. 8. Salicornia no. 2. 1768 ≡ *Arthrocnemum perenne* (Mill.) Moss ex Fourc., Mem. Bot. Surv. South Africa 20: 20. 1941 ≡ *S. fruticosa* var. *perennis* (Mill.) Fiori, Nuov. Fl. Italia 1: 426. 1923 ≡ *Sarcocornia perennis* (Mill.) A.J. Scott, Bot. J. Linn. Soc. 75: 367. 1978.

Neotype (designated by Guilló et al. [58] (p. 333)):—GREAT BRITAIN. England, Kent, Isle of Sheppey, c. 1850, *Thompson Lowne*” (K000450665!, image available in Guilló et al. [57] (p. 333)).

= *Salicornia perennis* var. *caespitosa* Rouy, Fl. France [Rouy and Foucaud] 12: 60. 1910.

Type: not designated.

= *Salicornia fruticosa* var. *deflexa* Rouy, Fl. France [Rouy and Foucaud] 12: 60. 1910.

Lectotype (designated here):—FRANCE. Normandy, *Saint-Vaast (Manche)*, *vases saleés*, 23 october 1887, *Corbière s.n.* (LY0745272!, image of the lectotype available at https://explore.recolnat.org/occurrence/E1A5EA9FAB934A2483203D1A0FCE7A94, accessed on 22 June 2024).

= *Salicornia radicans* Sm., Engl. Bot. 24: t. 1691. 1807.

Lectotype (designated here):—GREAT BRITAIN. England, coast of Sussex, 1805, *Borrer s.n.* (LINN-HS20-6!, image available at https://linnean-online.org/29500/#?s=0&cv=0&z=0.1996%2C0.7759%2C0.0986%2C0.1254, accessed on 22 June 2024).

= *Salicornia lignosa* J.Woods, Bot. Gaz. (London) 3(27): 31. 1851.

**Figure 8:**
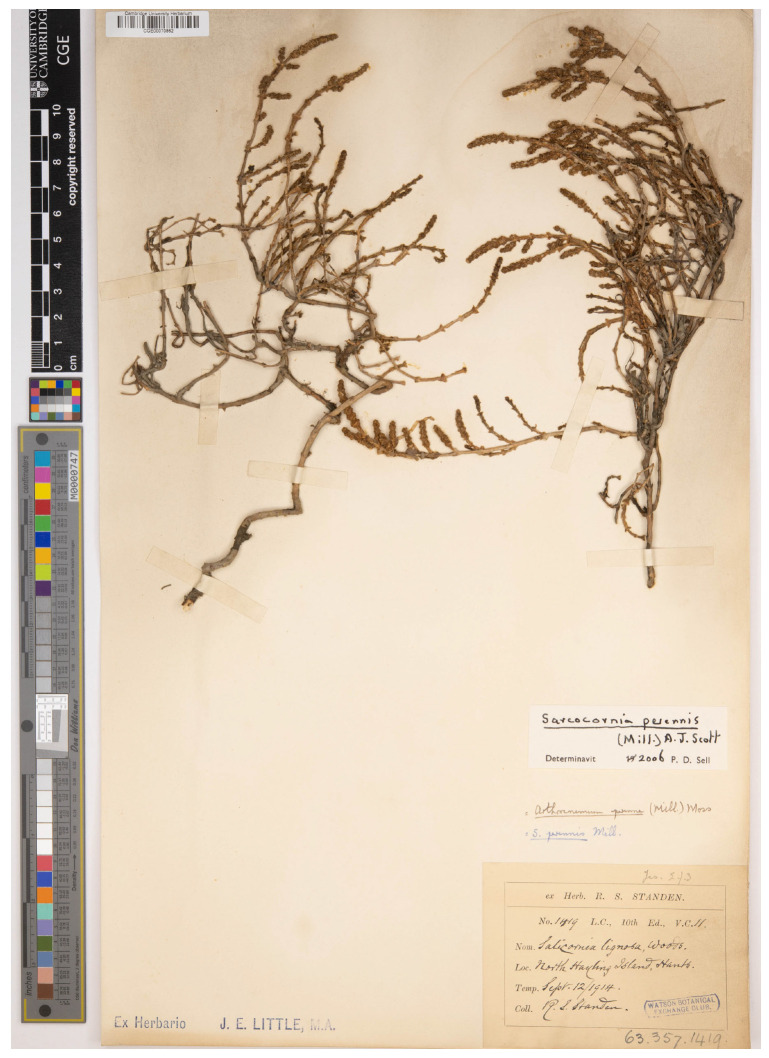
Neotype of *Salicornia lignosa* (CGE00070862!).

Neotype (designated here):—GREAT BRITAIN. England, North Hayling island, *Hants*, 12 September 1914, *R. S. Standen 1419* (CGE00070862!, Figure 8).

− *Salicornia fruticosa* sensu Sm., Engl. Bot. 35: Table 2467; image of the table available at https://archive.org/details/bim_eighteenth-century_english-botany-or-colo_smith-sir-james-edward_1813_35/page/n85/mode/2up, accessed on 22 June 2024.
− *Salicornia sempervirens* Sauvages ex Steud., Nomencl. Bot. [Steudel] 1: 714 (1821, *nom. inval.* (Art. 36.1a).

The following infraspecific names cannot currently be attributed to current taxa and are not synonymized here:

*Salicornia fruticosa* L. var. *intermedia* Ten., Syll. Pl. Fl. Neapol.: 582. 1831.

Neotype (designated here)—ITALY. Campania, luoghi salsi presso il lago Fusaro (Napoli), 28 settembre 1911, *Pellanda s.n.* (LY0517535!, image available at https://explore.recolnat.org/occurrence/E4E59CDB0F554CECB8594923690C7C81, accessed on 22 June 2024; isoneosyntype: LY0517536!, image at https://explore.recolnat.org/occurrence/B9EC0147368C4BB7AE376249A5CB7513, accessed on 22 June 2024).

*Salicornia fruticosa* L. var. *humilis* Ten., Syll. Pl. Fl. Neapol.: 582. 1831.

**Figure 9:**
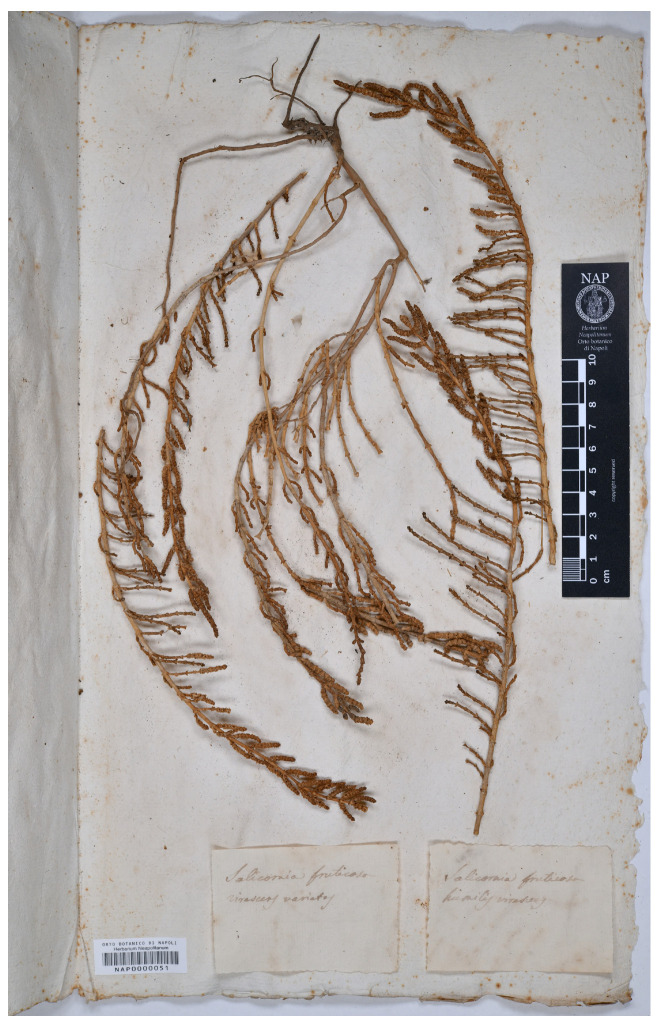
Neotype of *Salicornia fruticosa* var. *humilis* (NAP0000051!).

Neotype (designated here)—ITALY. *Sine locus*, *s.d.*, *Tenore s.n.* (NAP0000051!, part of the plant on the center of the sheet; Figure 9).

*Salicornia sarmentosa* Duval-Jouve, Bull. Soc. Bot. France 15: 174. 1869.

**Figure 10:**
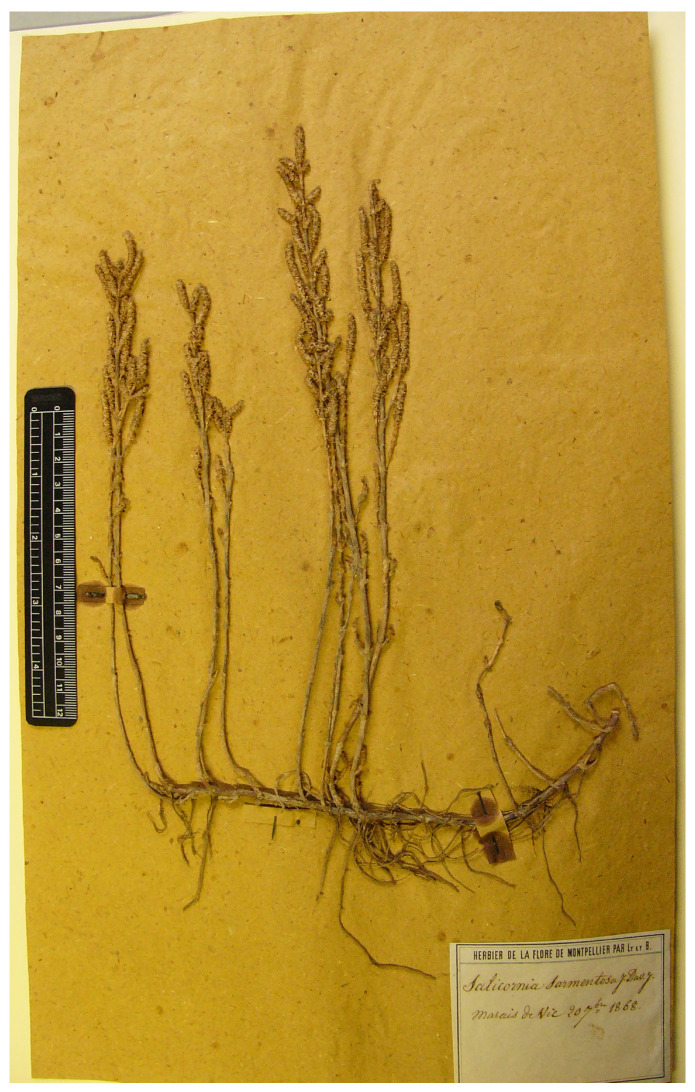
Lectotype of *Salicornia sarmentosa* (MPU!).

Lectotype (designated here):—FRANCE. Hérault, marais de Vic (HERBIER DE LA FLORE DEL MONTPELLIER), 20 Septembre 1868, *s.c.* [Lt. (Loret) and B, (Barrandon)] *s.n.* (MPU1316700!, Figure 10).

## Data Availability

Data are contained within the article.
